# Dickkopf1 as a Potential Biomarker in Polycystic Ovary Syndrome and Insulin Resistance: A Cross‐Sectional Study

**DOI:** 10.1111/1753-0407.70168

**Published:** 2025-11-30

**Authors:** Jiaxiu Ling, Hao Wang, Rui Liu, Juan Xiao, Sheng Qiu, Xiaotian Lei, Mengliu Yang, Yerui Lai, Hua Huang, Zerong Liang

**Affiliations:** ^1^ Department of Endocrinology, the Second Affiliated Hospital Chongqing Medical University Chongqing China; ^2^ Department of Endocrinology Chongqing Red Cross Hospital (People's Hospital of Jiangbei District) Chongqing China

**Keywords:** biomarker, cytokine, Dickkopf1, insulin resistance, polycystic ovary syndrome

## Abstract

**Background:**

Dickkopf1 (DKK1) is a protein with established links to metabolic diseases. However, its association with polycystic ovary syndrome (PCOS) and insulin resistance (IR) remains ambiguous.

**Methods:**

We conducted a cross‐sectional study involving 300 participants, including 100 healthy women, 100 women with PCOS, and 100 individuals with IR. Protein interactions with DKK1 were identified using the STRING database, followed by KEGG and GO analyses to explore enriched biological processes. Serum DKK1 and Adipoq levels were measured using ELISA kits. The hepatic DKK1 was detected by western blotting.

**Results:**

Both IR and PCOS groups showed significantly higher serum DKK1 levels and lower Adipoq levels compared to controls. Serum DKK1 levels positively correlated with body mass index (BMI), waist‐hip ratio (WHR), body fat percentage (FAT%), systolic blood pressure (SBP), triglycerides (TG), fasting plasma glucose (FPG), fasting insulin (FIns), glycosylated hemoglobin (HbA1c), homeostasis model assessment of insulin resistance (HOMA‐IR). Adipoq levels negatively correlated with glucose disposal rate (M‐value). Multiple regression analysis showed that BMI and Adipoq were independent factors affecting DKK1. Multiple stepwise regressions indicated that DKK1 is a risk factor for IR and PCOS. In the euglycemic‐hyperinsulinemic clamp (EHC) test, serum DKK1 levels increased in the PCOS patients and decreased in the IR patients at 30 min and returned to baseline at 60 min.

**Conclusions:**

Elevated DKK1 levels are strongly associated with PCOS and IR, suggesting a potential role in their development. This insight paves the way for further investigations into the role of DKK1 in PCOS and IR.

## Summary

- DKK1 are significantly elevated in women with PCOS and IR.
- A strong inverse correlation exists between circulating DKK1 and adiponectin levels, suggesting their opposing roles in metabolic regulation.
- We propose DKK1 as a potential negative regulator of insulin sensitivity and a novel biomarker for metabolic disturbances in PCOS.
- This cross‐sectional study identifies a need for further research to establish the causal relationship between DKK1 and PCOS‐related metabolic morbidities.

## Graphical abstract

The levels of DKK1 in patients with PCOS and IR are significantly elevated, revealing a potential relationship between DKK1, PCOS, and metabolic disorders, and suggesting that DKK1 may serve as a biomarker associated with PCOS and metabolic disorders.

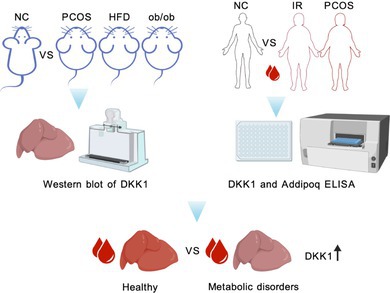

**Abbreviations**
Adipoq: adiponectin
BMI: body mass index
EHC: euglycemic‐hyperinsulinemic clamp
ELISA: enzyme‐linked immunosorbent assay
FPG: fasting plasma glucose
HOMA‐IR: homeostasis model assessment of insulin resistance
IR: insulin resistance
OGTT: oral glucose tolerance tests
SBP: systolic blood pressure
TG: triglyceride
WHR: waist hip ratio

## 1 Introduction

Polycystic ovary syndrome (PCOS) detrimentally impacts the health and fertility of women. Affecting 6%–10% of those of reproductive age, it is characterized by hyperandrogenism, persistent anovulation, and polycystic ovarian morphological changes [1]. The etiology of PCOS involves complex interactions of genetic and environmental factors. Obesity and insulin resistance (IR) are considered primary causes of reproductive dysfunction and metabolic abnormalities in PCOS patients, with approximately 50% of patients having variable degrees of IR. Therefore, improving obesity and IR is beneficial in enhancing reproductive function and reducing complications in women with PCOS. More recently, omics‐based approaches integrating genomics, transcriptomics, proteomics, and metabolomics have further broadened our understanding of the complex molecular networks underlying PCOS [2]. Recent investigations have also identified correlations between circulating cytokines and peptide hormones, such as adiponectin (Adipoq) and betatrophin, and PCOS, making it clinically pertinent to investigate the function of cytokines/peptide hormones in the genesis and progression of PCOS [3].

Dickkopf1 (DKK1) belongs to the DKK gene family, encompassing DKK1‐4. As a low molecular weight secretory glycoprotein, DKK1 is widely expressed across various organs, including osteoblasts, skin, prostate, placenta, and endothelial cells [1, 4]. DKK1 functions as an inhibitor of the Wnt/β‐catenin signaling pathway, participating in the pathogenesis of diverse diseases, such as cancer, hypertension, neurodegenerative disorders, diabetic nephropathy, and arthritis [5, 6]. Recent findings have reported elevated DKK1 levels in individuals with obesity and those with type 2 diabetes mellitus (T2DM), displaying a positive correlation with body mass index (BMI) [7].

DKK1 expression increases during fat analysis but is lower in mature adipocytes, indicating its potential relationship with obesity [8]. However, the connection between DKK1, PCOS, and IR remains ambiguous.

In the current investigation, we examined the involvement of DKK1 in the pathogenesis of PCOS and IR using a combination of bioinformatics analysis, a population‐based cross‐sectional study, and multiple intervention studies. Our results offer empirical support to assess the potential of DKK1 as an early diagnostic biomarker for PCOS.

## 2 Materials and Methods

### 2.1 Study Population

This study encompassed 300 young women (aged 16–35 years) from the Second Affiliated Hospital of Chongqing Medical University, consisting of 100 patients with PCOS, 100 patients with IR, and 100 healthy controls. All participants, who were outpatients, underwent physical examinations. IR was diagnosed employing a homeostasis model assessment index (HOMA‐IR) > 3.0 [9], with no presence of PCOS or other metabolic diseases. The Rotterdam criteria, updated in 2003 [10], served as the diagnostic basis for PCOS patients. Exclusion criteria: (1) Major organ dysfunction: Renal insufficiency (eGFR < 60 mL/min/1.73m^2^); hepatic insufficiency (ALT or AST > 3 × ULN or Child‐Pugh class B/C); Thyroid dysfunction (TSH < 0.1 mIU/L or > 10 mIU/L). (2) Metabolic/endocrine‐interfering treatments: Use of oral contraceptives, glucocorticoids, or insulin‐sensitizing agents (metformin/thiazolidinediones) within 6 months; Use of androgen inhibitors (e.g., spironolactone) within 3 months. (3) Severe comorbidities: Cardiovascular disease (NYHA class III/IV heart failure, myocardial infarction/stroke within 6 months); Active infectious diseases (HIV, HBV, HCV, tuberculosis); Malignancy (diagnosed or under treatment within 5 years). (4) Severe psychiatric disorders: depression/schizophrenia requiring hospitalization. Informed consent was obtained from all participants. This study was approved by the Human Research Ethics Committee of Chongqing Medical University (2014 Ethical Review No. 72) and registered at the Chinese Clinical Trial Registry (ChiCTR2000032878).

Liver samples were obtained from 4 patients with NAFLD and 4 non‐NAFLD controls who underwent liver biopsy or hepatic surgery. Exclusion criteria included excessive alcohol consumption (> 140 g/week for men, > 70 g/week for women), history of drug/toxin exposure, and viral infections (HBV/HCV). All procedures involving human samples were approved by the Ethics Committee of Chongqing Medical University (Approval No. 2015 Ethical Review No. 74), and written informed consent was obtained in accordance with the Declaration of Helsinki. Pathological classification was based on the NAFLD Activity Score (NAS): cases with NAS ≥ 3 were defined as NAFLD, whereas cases with NAS = 0 served as non‐NAFLD controls.

### 2.2 Bioinformatics Analysis

To establish a protein–protein interaction (PPI) network centered on the DKK1 gene, we utilized the STRING database (version 11.5). We applied a threshold interaction score of 0.4 as the cutoff criterion and subsequently visualized the PPI network. To further decipher functional annotations and pathways, we conducted gene ontology (GO) and Kyoto Encyclopedia of Genes and Genomes (KEGG) pathway analyses using the clusterProfiler package [11]. To further validate the key interactions predicted by the PPI network, molecular docking simulations were performed between DKK1 and two high‐confidence partners, LRP6 and KREMEN1. The three‐dimensional structure of DKK1 was obtained from the Protein Data Bank (PDB ID: 3S2K), and the structures of LRP6 and KREMEN1 were retrieved from the AlphaFold database. Docking simulations were conducted using the ZDOCK module in Discovery Studio. The binding affinities of the resulting complexes were evaluated by calculating their binding energies (kcal/mol) with the PDBePISA online server.

### 2.3 Anthropometric Measurements

An experienced physician conducted standardized anthropometric measurements on all participants, including body weight, height, waist circumference (WC), hip circumference, blood pressure, and fat percentage (Fat%), performed by a single, experienced physician. BMI was calculated as weight (kg) divided by height squared (m^2^). The waist‐hip ratio (WHR) was calculated as WC (cm) divided by HC (cm). After a 12‐h overnight fast, professional nurses collected venous blood samples to measure blood glucose, insulin, HbA1c, blood lipids, sex hormones, and other indicators. HOMA‐IR was computed using the following formula: fasting insulin (FIns, μU/L) × fasting blood glucose (FBG, mmol/L) divided by 22.5 [12].

### 2.4 Determination of Biochemical Indexes and Sex Hormones

FBG, HbA1c, serum insulin, triglycerides (TG), cholesterol (TC), free fatty acids (FFAs), high‐density lipoprotein cholesterol (HDL‐C), and low‐density lipoprotein cholesterol (LDL‐C) were measured as previously described [13]. Sex hormone assays, encompassing luteinizing hormone (LH), follicle‐stimulating hormone (FSH), progesterone (Prog), testosterone (TEST), sex hormone binding globulin (SHBG), estradiol (E2), and dehydroepiandrosterone sulfate (DHEA‐S), were performed in accordance with previously published methodologies [13].

### 2.5 Oral Glucose Tolerance Test (OGTT)

Following a 12‐h nocturnal fasting period, participants were subjected to an OGTT. Participants were administered 75 75 g glucose orally, and their blood glucose, insulin, and DKK1 levels, as well as other biomarkers, were assessed at 0, 30, 60, and 120‐min time points using venous blood samples, in accordance with the methodology outlined in the reference [14].

### 2.6 Euglycemic‐Hyperinsulinemic Clamp (EHC)

A total of 40 PCOS patients, 39 IR patients, and 40 healthy controls underwent the EHC test as previously reported [15]. After fasting for 12 h, insulin and glucose were infused through catheters in the anterior cubital vein and the opposite dorsal hand vein, respectively. An insulin infusion rate of 1 mU/kg/min was maintained for 2 h, accompanied by a simultaneous infusion of 20% glucose. Blood glucose levels were stabilized at basal concentrations by adjusting the glucose infusion rate. Insulin and DKK1 levels were evaluated using blood samples collected at 0, 80, 100, and 120 min.

### 2.7 Serum Cytokine Determination

Serum levels of DKK1 and Adipoq were measured using highly sensitive and specific ELISA kits, exhibiting negligible cross‐reactivity. The intra‐ and inter‐assay coefficients of variation (CVs) for DKK1 were < 10% and < 12%, respectively, with a detection range of 100–30 000 pg/mL. Intra‐ and inter‐assay CVs for Adipoq were as documented in a prior study [15].

### 2.8 Experimental Animals

Male C57BL/6J (8‐week‐old) and ob/ob mice were acquired from the Experimental Animal Centers of the Model Animal Research Center at Nanjing University. Following a 7‐day acclimatization period at room temperature (25°C), mice were fed either a normal chow diet (NCD) or a high‐fat diet (HFD; carbohydrate, 20%; fat, 60%; protein, 20%; D12492, Research Diets, New Brunswick, NJ) for 12 weeks. Liver tissues were collected and preserved at −180°C until further analysis. The animal experiments received approval from the Animal Studies Committee of The Second Affiliated Hospital of Chongqing Medical University (IACUC‐SAHCQMU‐2024‐00072) and were conducted in accordance with the National Institutes of Health Guide for the Care and Use of Laboratory Animals (NIH Publications No. 8023, revised 1978). PCOS model mice were obtained from Chongqing Yuanyuan Weier Biotechnology Co. Ltd. (Chongqing, China).

### 2.9 Western Blot Analysis of Proteins

Liver tissue‐derived total proteins underwent sodium dodecyl sulfate–polyacrylamide gel electrophoresis (SDS–PAGE) and subsequent transfer onto polyvinylidene difluoride (PVDF) membranes. These membranes were incubated with anti‐DKK1 antibody (1:1000, #48367, Cell Signaling Technology, USA) and GAPDH (1:1000, #5174, Cell Signaling Technology, USA) as internal controls. Horseradish peroxidase‐conjugated secondary antibodies (1:2000, #A0208, Beyotime, China) were subsequently applied. Densitometric analyses were conducted using ImageJ software in accordance with previously published methods [16].

### 2.10 Statistical Analysis

Analysis employed SPSS software (v20.0, Chicago, IL, USA), with graphs generated through GraphPad software. Data are presented as the mean ± standard deviation (mean ± SD). For data with a normal distribution, independent samples t‐tests or ANOVA were employed for group comparisons, while the Mann–Whitney U test or Kruskal–Wallis H test was employed for nonnormally distributed data. Correlations between DKK1 and other variables were analyzed using simple and multiple correlation coefficients. Prior to performing multivariate regression analyses, multicollinearity among independent variables was assessed using the variance inflation factor (VIF); a VIF value exceeding 10 was considered indicative of significant multicollinearity. Binary logistic regression analyses, the row mean score test, and the Cochran‐Armitage trend test were implemented to investigate the association between circulating DKK1 and PCOS and IR. Receiver operating characteristic (ROC) curves generated by SPSS 20.0 were used to assess the sensitivity and specificity of DKK1 for predicting PCOS and IR. A *p* value < 0.05 indicated statistical significance.

## 3 Results

### 3.1 Bioinformatics Analysis

**FIGURE 1:**
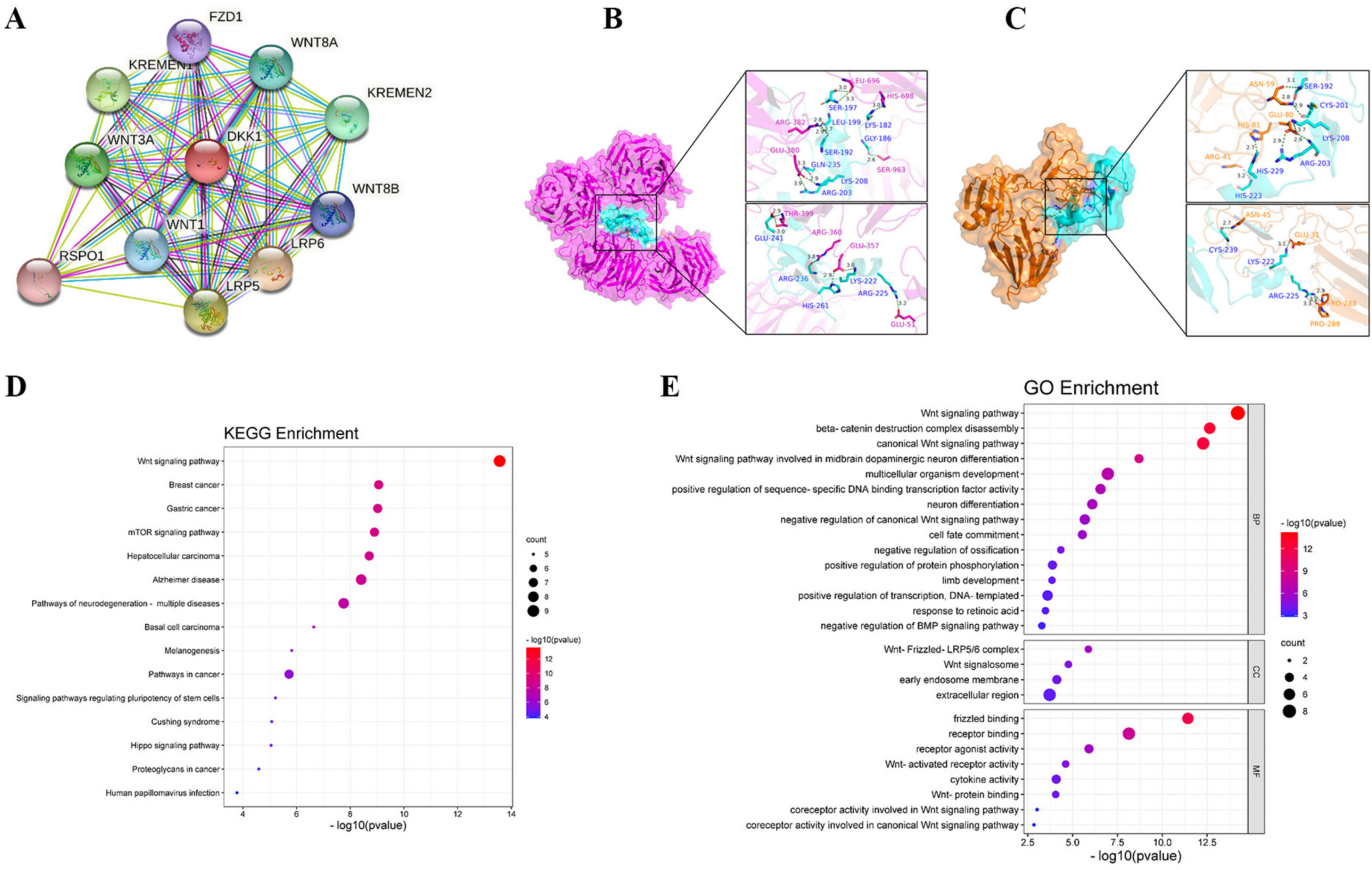
Bioinformatics analysis related to DKK1 and associated signaling pathways. (A) PPI network related to DKK1. (B) Docking model of DKK1 (cyan) and LRP6 (purple). (C) Docking model of DKK1 (cyan) and KREMEN1 (orange). (D) The enriched pathways of the KEGG analysis. (E) The results of GO enrichment analysis. The x‐axis represents the ratio of involved genes, and the y‐axis represents the GO and KEGG terms. Each bubble represents a term. A larger bubble indicates more involved genes. Red color indicates smaller *p* values. GO, gene ontology; KEGG, Kyoto Encyclopedia of Genes and Genomes.

To investigate the relationship between DKK1 and metabolism, we conducted a preliminary bioinformatics analysis using online data. We constructed a PPI network for DKK1 utilizing the STRING database. Our findings reveal that DKK1 interacts with a multitude of proteins, such as LRP6, LRP5, KREMEN1, WNT1, WNT3A, WNT8B, WNT8A, KREMEN2, FZD1, and RSPO1, among others (Figure 1A). To validate these predicted interactions, we performed molecular docking simulations between DKK1 and two high‐confidence partners, LRP6 and KREMEN1. The docking results demonstrated stable binding conformations with favorable binding energies, supporting the plausibility of these molecular interactions (Figure 1B,C). We subsequently performed GO and KEGG enrichment analyses using the DAVID tool, which indicated that DKK1‐related signaling pathways included Wnt signaling, Wnt‐protein binding, cytokine activity, multicellular organism development, negative regulation of protein phosphorylation, mTOR signaling, and pathways in cancer (Figure 1D,E). These findings suggest that DKK1 may influence metabolic regulation through interaction with Wnt‐related proteins and associated signaling pathways.

### 3.2 Elevated Hepatic DKK1 Expression in Metabolic Disease Murine Models and in NAFLD Patients

**FIGURE 2:**
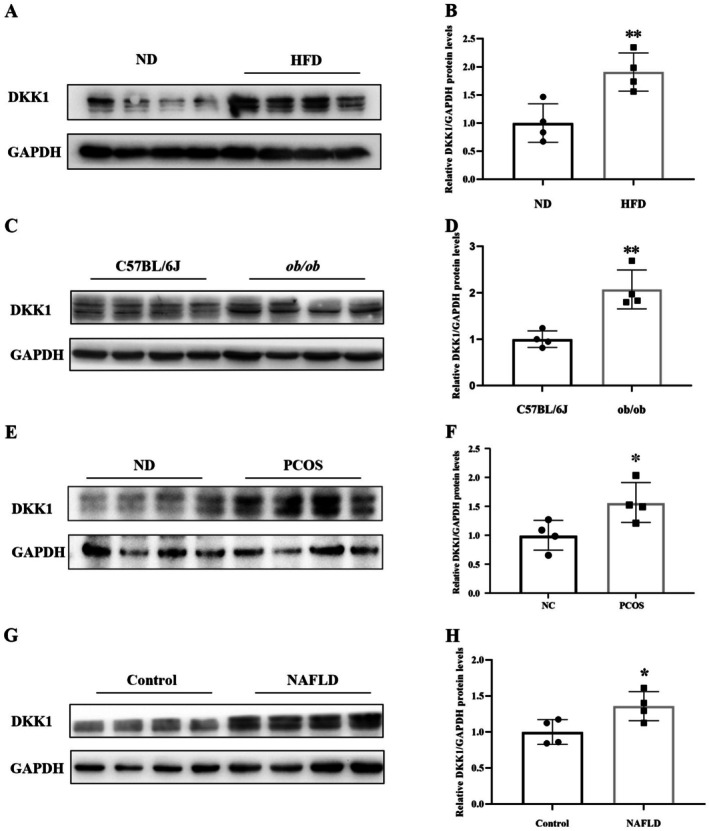
DKK1 protein expression in the liver of HFD‐fed and obese mice and humans. (A and B) DKK1 protein expression in the livers of ND‐ and HFD‐fed mice. (C and D) DKK1 protein expression in the livers of C57BL/6J and ob/ob mice. (E and F) DKK1 protein expression in the livers of ND and *PCOS* mice. (G and H) DKK1 protein expression in the livers of NAFLD patients and normal individuals. ND, normal chow diet; HFD, high‐fat diet; NAFLD, nonalcoholic fatty liver disease. Data are mean ± SD, *n* = 3–5 for each group. *p < 0.05 or **p < 0.01 versus controls.

To elucidate the link between DKK1 and metabolic perturbations, we scrutinized DKK1 expression profiles in murine models of obesity, diabetes and PCOS, as well as in human subjects diagnosed with NAFLD. Utilizing western blot analyses, we observed a marked upregulation of hepatic DKK1 protein expression in HFD‐fed WT mice, ob/ob mice and PCOS mice, relative to their ND‐fed WT counterparts (Figure 2A–F). Similarly, DKK1 protein expression in the livers of NAFLD patients was substantially elevated compared to that observed in normal control subjects (Figure 2G,H). These findings further support the notion that DKK1 is associated with metabolic diseases.

### 3.3 Clinical Features of the Study Population

**FIGURE 3:**
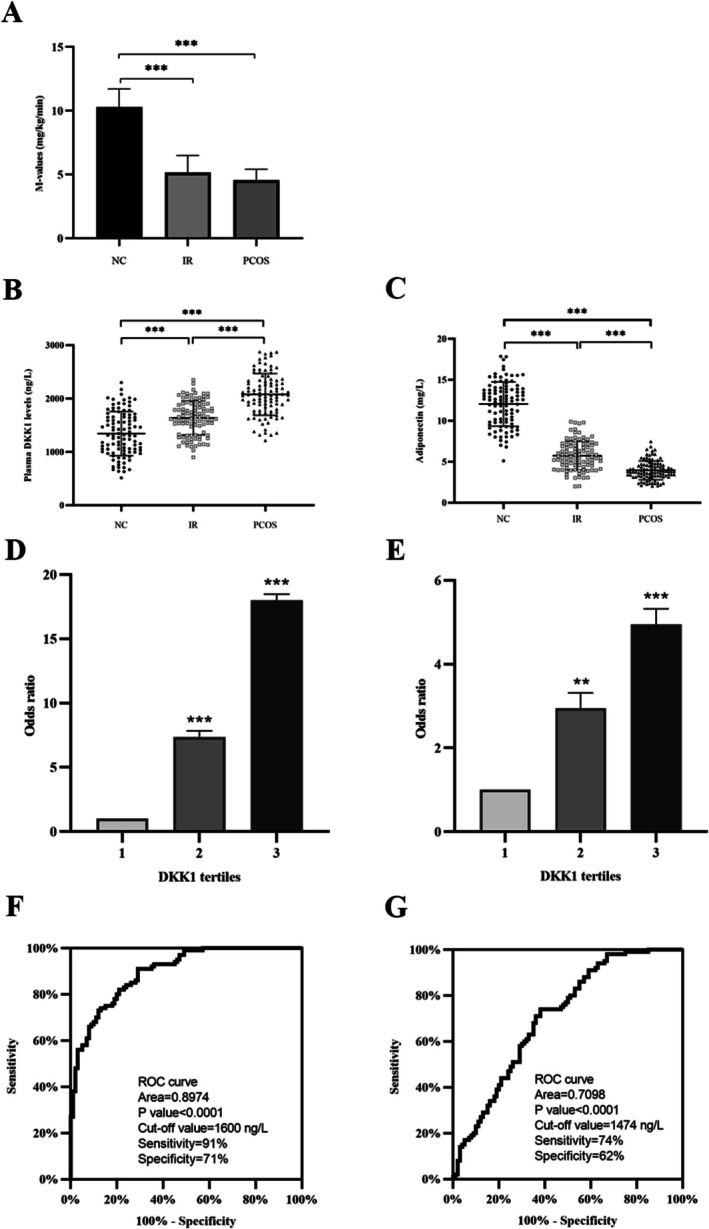
Insulin clamp M‐values and circulating cytokine levels in the study population. (A) M‐values in all study subjects during the EHC. (B) Serum DKK1 levels in all study populations. (C) Serum adiponectin levels in all participants. (D and E) The prevalence of elevated PCOS (D) and IR (E) in different tertiles of DKK1: Tertile 1, < 1.28 μg/L; tertile 2, 1.28 to 1.71 μg/L; and tertile 3, > 1.71 μg/L for IR; tertile 1, < 1.46 μg/L; tertile 2, 1.46 to 1.98 μg/mL; and tertile 3, > 1.98 μg/L for PCOS. (F and G) Receiver operating characteristic curve (ROC) analysis for the prediction of PCOS (F) and IR (G) according to DKK1 levels. Data are presented as the mean ± SD; *p < 0.05, **p < 0.01.

Table 1 displays a significant elevation in BMI, WHR, FAT%, SBP, TGs, TC, LDL‐C, FBG, OGTT blood glucose, FIns, OGTT insulin, HbA1c, and HOMA‐IR at each respective time point within the IR and PCOS patients compared to the control group. Concurrently, the insulin sensitivity index (M‐values) exhibited a notable decline (Figure 3A). Additionally, analysis of sex hormones revealed that DHEA‐S levels in both the PCOS and IR groups were considerably elevated compared to the control group, while SHBG levels were markedly reduced. Moreover, the PCOS group exhibited significantly increased TEST and LH levels relative to the control group (Table 1). These findings indicate the presence of hormonal imbalances in both the PCOS and IR groups, with PCOS women exhibiting a distinct hyperandrogenemic profile.

**TABLE 1:** Main clinical features, laboratory parameters and circulating DKK1 concentrations in the study population.

Variable	NC (*n* = 100)	IR (*n* = 100)	PCOS (*n* = 100)
Age (years)	26.97 ± 2.83	27.39 ± 3.40	26.71 ± 3.88
BMI (kg/m^2^)	20.71 ± 2.53	25.34 ± 2.61**	26.11 ± 3.26**
WHR	0.78 ± 0.04	0.85 ± 0.05**	0.86 ± 0.06**
FAT (%)	26.37 ± 4.43	35.52 ± 5.12**	34.93 ± 5.92**
SBP (mmHg)	107.77 ± 7.01	119.17 ± 14.72**	117.48 ± 12.01**
DBP (mmHg)	75.11 ± 7.49	77.52 ± 10.93	74.63 ± 9.02
TG (mmol/L)	0.98 ± 0.59	0.91 ± 0.52	1.64 ± 0.97**
TC (mmol/L)	3.49 ± 1.31	3.75 ± 1.21	4.48 ± 0.98**
HDL‐C (mmol/L)	1.23 ± 0.41	1.26 ± 0.28	1.31 ± 0.37
LDL‐C (mmol/L)	2.05 ± 0.89	2.62 ± 0.68**	2.57 ± 0.66**
FFA (mmol/L)	0.54 ± 0.28	0.56 ± 0.16	0.54 ± 0.21
FBG (mmol/L)	4.51 ± 0.49	5.45 ± 0.62**	5.32 ± 0.80**
0.5 h‐BG (mmol/L)	7.25 ± 1.46	9.60 ± 1.72**	9.00 ± 1.61**
1 h‐BG (mmol/L)	5.82 ± 1.47	9.49 ± 2.37**	9.20 ± 2.50**
2 h‐BG (mmol/L)	5.10 ± 0.94	7.96 ± 1.83**	7.81 ± 2.26**
FIns (mU/L)	7.71 ± 2.65	19.89 ± 9.25**	19.18 ± 13.73**
0.5 h‐Ins (mU/L)	91.82 ± 58.77	149.25 ± 110.39**	130.08 ± 81.84**
1 h‐Ins (mU/L)	72.10 ± 70.87	178.71 ± 165.10**	154.09 ± 115.00**
2 h‐Ins (mU/L)	42.90 ± 36.95	151.05 ± 104.16**	161.58 ± 142.30**
HOMA‐IR	1.56 ± 0.59	4.88 ± 2.63**	4.69 ± 4.04**
HbA1c (%)	5.10 ± 0.25	5.31 ± 0.44**	5.45 ± 0.48**
Adiponectin (mg/L)	12.04 ± 2.69	5.76 ± 1.74**	3.96 ± 1.14**
M value (mg/kg/min)	10.29 ± 1.42	5.15 ± 1.33**	4.56 ± 0.85**
DHEA‐S (μg/dL)	196.69 ± 66.87	241.95 ± 95.67**	263.91 ± 136.89**
SHBG (nmol/L)	57.99 ± 23.81	43.00 ± 27.35**	37.80 ± 24.42**
E2 (ng/L)	50.58 ± 28.49	47.88 ± 25.82	49.70 ± 32.01
TEST (nmol/L)	1.71 ± 0.64	1.60 ± 0.59	2.00 ± 0.81**
FSH (IU/L)	7.30 ± 1.58	6.83 ± 2.33	6.78 ± 1.71*
LH (IU/L)	5.27 ± 2.77	4.88 ± 2.92	7.59 ± 2.51**
Prog (IU/L)	2.25 ± 1.25	2.05 ± 1.04	2.13 ± 1.33
PRL (mIU/L)	318.52 ± 114.28	318.74 ± 141.99	302.10 ± 137.94
DKK1 (μg/L)	1.34 ± 0.41	1.64 ± 0.32**	2.08 ± 0.39**

*Note:* Results and corresponding *p* value for correlations between main clinical features, laboratory parameters and circulating DKK1 concentrations in the study population. Values are given as the means ± SDs. *p < 0.05; *p < 0.01, compared with the NC group.

Abbreviations: 2 h‐BG, 2 h blood glucose overload; 2 h‐Ins, 2 h plasma insulin after glucose overload; BMI, body mass index; DBP, diastolic blood pressure; DHEA‐S, dehydroepiandrosterone sulfate; E2, Estradiol; FBG, fasting blood glucose; FFA, free fatty acid; FIns, fasting plasma insulin; FPG, fasting plasma glucose; FSH, follicular stimulating hormone; HbA1c, glycosylated hemoglobin; HDL‐C, high‐density lipoprotein cholesterol; HOMA‐IR, homeostasis model assessment of insulin resistance; IR, insulin resistance; LDL‐C, low‐density lipoprotein cholesterol; LH, luteinizing hormone; NC, normal control; PCOS, polycystic ovary syndrome; PRL, prolactin; Prog, progesterone; SBP, systolic blood pressure; SHBG, sex hormone‐binding globulin; TC, total cholesterol; TEST, testosterone; TG, triglyceride; WHR, waist‐hip ratio.

### 3.4 Circulating Cytokine Levels and Their Relationship With Other Indexes

To elucidate the interplay between DKK1, metabolic disorders, and IR in PCOS patients, we concurrently measured the levels of circulating DKK1 and Adipoq, an insulin‐sensitive molecule. Our findings indicated a significant elevation in circulating DKK1 levels accompanied by a substantial decrease in Adipoq levels in women with both PCOS and IR compared to healthy control subjects (Table 1 and Figure 3B,C). Notably, DKK1 levels were significantly higher in PCOS patients than in IR individuals (Figure 3B). We also employed linear correlation analysis to assess the association between serum DKK1 and Adipoq, as well as other indicators. Our analysis demonstrated a significant positive correlation between serum DKK1 and BMI, WHR, Fat%, SBP, TGs, blood glucose, FIns, and HbA1c but a negative correlation with Adipoq (Table 2). Moreover, multiple regression analysis identified BMI, fat%, TGs, and Adipoq as independent risk factors influencing serum DKK1 levels (Table 2). The resulting regression equation was Y_DKK1 = 0.134 + 0.022BMI + 0.017FAT + 0.227TG—0.024Adipoq.

**TABLE 2:** Linear and multiple regression analyses of variables associated with serum DKK1 levels in the study population.

Variable	Simple		Multiple	
	*r*	*p*	*b*	*p*
Age (years)	−0.022	0.701	—	—
BMI (kg/m2)	0.529	< 0.001	0.169	0.05
WHR	0.404	< 0.001	—	—
FAT (%)	0.502	< 0.001	0.231	0.004
SBP (mmHg)	0.212	< 0.001	—	—
DBP (mmHg)	−0.011	0.847	—	—
TG (mmol/L)	0.604	< 0.001	0.373	< 0.001
TC (mmol/L)	0.041	0.477	—	—
HDL‐C (mmol/L)	−0.027	0.642	—	—
LDL‐C (mmol/L)	0.038	0.512	—	—
FFA (mmol/L)	0.018	0.761	—	—
FPG (mmol/L)	0.252	< 0.001	—	—
FIns (mU/L)	0.304	< 0.001	—	—
HbA1c (%)	0.303	< 0.001	—	—
Adiponectin (mg/L)	−0.179	0.002	−0.201	< 0.001
DHEA‐S (μg/dL)	0.045	0.433	—	—
SHBG (nmol/L)	−0.059	0.312	—	—
E2 (ng/L)	−0.008	0.897	—	—
TEST (nmol/L)	0.107	0.064	—	—
LH (IU/L)	0.089	0.123	—	—
Prog (IU/L)	0.079	0.171	—	—
PRL (mIU/L)	−0.087	0.132	—	—

Abbreviations: *b*: regression coefficient; *r*: correlation coefficient.

### 3.5 Relationship of Circulating DKK1 With PCOS and IR

To further explore the link between DKK1 and PCOS and IR, we separated DKK1 levels into three groups within the study population. For IR, the groups were as follows: group 1, < 1.284 μg/L; group 2, 1.285 to 1.71 μg/L; and group 3, > 1.71 μg/L. For PCOS, the groups were defined as follows: group 1, < 1.46 μg/L; group 2, 1.47 to 1.98 μg/L; and group 3, > 1.98 μg/L. Our analysis revealed that when serum DKK1 concentrations were within tertiles 2 and 3, the odds ratios (ORs) for the presence of PCOS and IR were 7.36 [95% confidence interval (CI), 2.90; 18.69] and 18.01 [95% CI, 7.08; 45.77] for PCOS and 2.95 [95% CI, 1.44; 6.04] and 4.95 [95% CI, 2.36; 10.36] for IR, respectively (Figure 3D,E). Multicollinearity diagnostics confirmed that all variance inflation factor (VIF) values for the independent variables in both the PCOS and IR models were well below the threshold of 10 (range: 1.12–4.16), indicating that multicollinearity was not a significant concern. Furthermore, a multivariable logistic regression analysis demonstrated a significant association between DKK1 levels and PCOS and IR after accounting for anthropometric variables, age, sex, fat%, BP, and other factors (Table 3). The row mean score difference and the Cochran–Armitage trend test also showed a significant linear trend of increasing DKK1 levels with PCOS and IR (Table 4). Finally, we conducted an ROC analysis, which indicated an area under the curve (AUC) of 0.897 with 91% sensitivity and 71% specificity for PCOS (Figure 3F) and an AUC of 0.71 with 74% sensitivity and 62% specificity for IR (Figure 3G).

**TABLE 3:** Association of serum DKK1 with IR and PCOS in the fully adjusted model.

Model adjust	IR			PCOS		
	OR	95% CI	*p*	OR	95% CI	*p*
Age, BMI	1.001	1.000–1.002	0.192	1.003	1.002–1.005	< 0.001
Age, BMI, WHR	1.001	1.000–1.002	0.221	1.003	1.002–1.005	< 0.001
Age, BMI, WHR, FAT%	1.000	0.999–1.001	0.714	1.003	1.002–1.005	< 0.001
Age, BMI, WHR, FAT%, SBP, DBP	1.000	0.999–1.001	0.719	1.003	1.002–1.005	< 0.001
Age, BMI, WHR, FAT%, SBP, DBP, lipid profile	1.004	1.002–1.007	0.001	1.004	1.002–1.006	< 0.001

*Note:* Multivariable logistic regression analysis demonstrated a significant association between DKK1 levels and PCOS and IR after accounting for anthropometric variables.

**TABLE 4:** Row mean scores and Cochran–Armitage trend test of the impact of serum DKK1 level on IR and PCOS.

Model adjusted	IR		PCOS	
	*χ* ^2^	*p*	*χ* ^2^	*p*
Row mean scores test	17.9630	< 0.001	82.4834	< 0.001
Cochran‐Armitage trend test	−4.2489	< 0.001	−9.1048	< 0.001

*Note:* The row mean score difference and the Cochran–Armitage trend test showed a significant linear trend of increasing DKK1 levels with PCOS and IR.

### 3.6 Validation of DKK1 as a Biomarker for PCOS and IR

**FIGURE 4:**
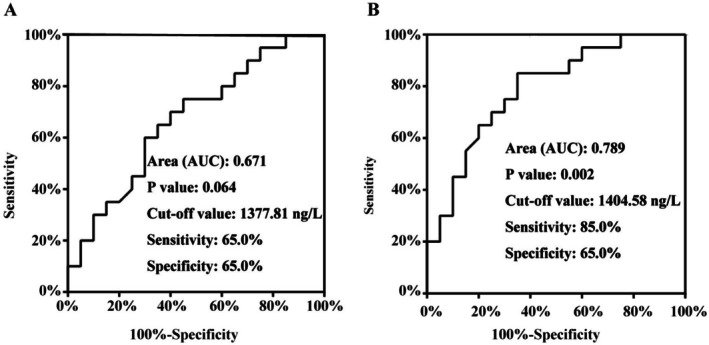
Validation of DKK1 as a Biomarker for PCOS and IR. (A) ROC curve demonstrating DKK1's ability to distinguish insulin resistance patients from healthy controls (AUC = 0.671, *p* = 0.064). (B) ROC curve showing DKK1's diagnostic performance for polycystic ovary syndrome identification (AUC = 0.789, *p* = 0.002). Optimal cut‐off values, sensitivity, and specificity are indicated for each analysis.

To validate our initial findings, we analyzed an independent cohort comprising 20 normal controls (NC), 20 IR patients, and 20 PCOS patients. Consistent with the primary cohort, serum DKK1 levels showed a stepwise increase from NC to IR to PCOS, with the highest concentrations observed in PCOS patients (Table 5). ROC curve analysis indicated that DKK1 had moderate diagnostic accuracy for PCOS (AUC = 0.789, *p* = 0.002), yielding 85.0% sensitivity and 65.0% specificity at an optimal cutoff of 1404.58 ng/L (Figure 4B). In contrast, the diagnostic performance for isolated IR was limited (AUC = 0.671, *p* = 0.064), with balanced sensitivity and specificity of 65.0% (Figure 4A). Importantly, ANCOVA confirmed that differences in DKK1 levels among groups remained statistically significant after adjusting for BMI (*p* = 0.003), suggesting that the association of DKK1 with PCOS is independent of obesity‐related factors.

**TABLE 5:** Main clinical features and circulating DKK1 concentrations in the study population.

Variable	NC (*n* = 100)	IR (*n* = 100)	PCOS (*n* = 100)
Age (years)	28.5 ± 3.2	30.1 ± 4.1	29.8 ± 3.5
BMI (kg/m^2^)	22.3 ± 1.8	26.7 ± 2.3**	28.1 ± 2.6**
DKK1 (μg/L)	1278.1 ± 558.4	1635.1 ± 622.4**	1932.2 ± 613.2**

*Note:* Results and corresponding *p* value for correlations between main clinical features and circulating DKK1 concentrations in the study population. Values are given as the means ± SDs. *p < 0.05; **p < 0.01, compared with the NC group.

### 3.7 Effect of Blood Glucose and Insulin Levels on DKK1

**FIGURE 5:**
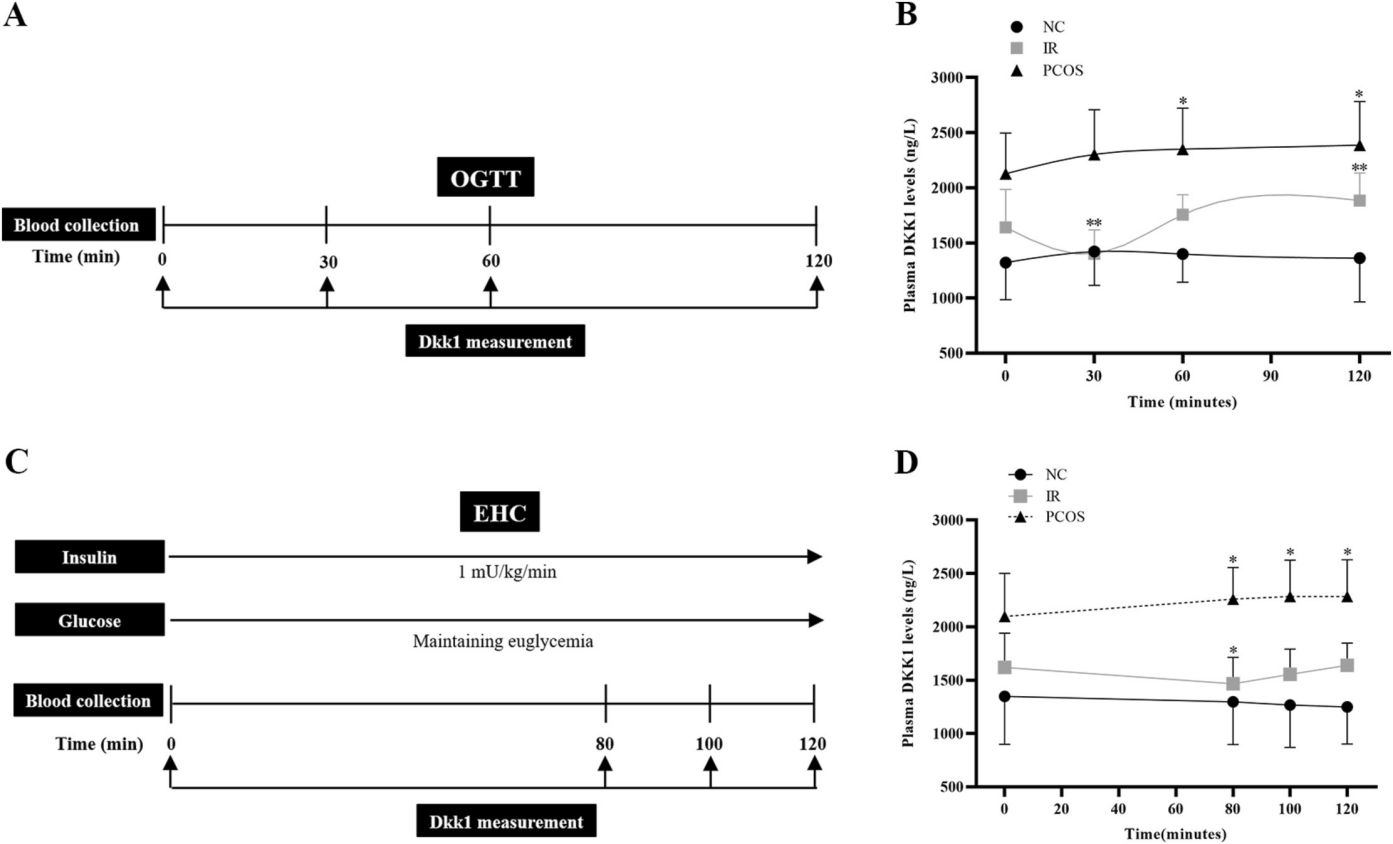
Circulating DKK1 levels in interventional studies. (A) Flowchart of OGTT in all participants. (B) Circulating DKK1 levels during the OGTT. (C) Flowchart of EHC in all subjects. (D) Circulating DKK1 levels during EHC. Data are presented as the mean ± SD; *p < 0.05 or **p < 0.01 vs. baseline.

To evaluate the potential impact of blood glucose and insulin levels on circulating DKK1 concentrations, we conducted an OGTT (Figure 5A). During the OGTT, we observed that serum DKK1 levels in PCOS patients steadily increased (from 2.13 ± 0.36 to 2.39 ± 0.39 μg/L) alongside rising blood glucose and insulin levels. On the other hand, in IR individuals, DKK1 levels significantly declined (from 1.64 ± 0.34 to 1.40 ± 0.21 μg/L) at 30 min and then incrementally rose (from 1.76 ± 0.18 to 1.88 ± 0.25 μg/L). In the normal control group, DKK1 levels remained constant throughout (Figure 5B). These results imply that circulating DKK1 might be influenced by blood glucose and/or insulin in women with PCOS and IR. To further explore whether insulin regulates DKK1, we performed an EHC to maintain blood glucose at basal levels while increasing insulin levels (Figure 5C). Throughout the EHC, blood glucose was kept within the range of 5–6 mmol/L, as insulin levels increased from 7.71 ± 2.65 to 80.5 ± 10.5 μU/L. Our results demonstrated that the DKK1 levels did not change in the normal control group compared to the baseline level under the conditions of normal blood glucose and hyperinsulinemia. In contrast, DKK1 levels in the PCOS group exhibited a significant increase (from 2.10 ± 0.40 to 2.29 ± 0.34 μg/L) compared to baseline levels. In contrast, DKK1 levels in the IR group decreased significantly at 30 min (from 1.62 ± 0.32 to 1.467 ± 0.24 μg/L) and returned to baseline levels at 60 min (Figure 5D). These results suggest that DKK1 levels may be influenced by both blood glucose and insulin in PCOS and IR individuals.

## 4 Discussion

Previous research has indicated a possible connection between DKK1 and obesity. Emerging evidence indicates that DKK1 contributes to PCOS pathophysiology through dual mechanisms: enhancing fatty acid uptake via the ERK–PPARγ–CD36 axis and inducing IR through JNK signaling [17]. In addition, DKK1 promotes deacetylation and activates the TGF‐β1/Smad3 pathway, thereby inhibiting proliferation and promoting apoptosis of ovarian granulosa cells, which exacerbates PCOS progression [18]. Clinical observations also show that circulating DKK1 levels are elevated in overweight/obese individuals and are associated with a higher prevalence of obesity [19]. These findings highlight DKK1 as a potential therapeutic target in PCOS. Recent multi‐omics analyses have further advanced our understanding of PCOS pathogenesis by integrating genomic, transcriptomic, proteomic, and metabolomic data to uncover key molecular networks and pathways [13]. Such approaches highlight the complexity of PCOS as a systemic metabolic and hormonal disorder and suggest that molecules like DKK1 may function as part of broader regulatory networks.

Notably, PCOS is a recognized metabolic and hormonal disorder [20], and the relationship between DKK1 and PCOS and IR remains unclear. To shed light on this relationship, we conducted a cross‐sectional population study using HOMA‐IR and EHC studies to compare IR in women with PCOS and control subjects. We found that women with PCOS exhibited more severe IR and significantly increased levels of DKK1, while Adipoq levels were significantly reduced. Additionally, we discovered that serum DKK1 levels demonstrated a positive correlation with clinical markers of obesity, biochemical indicators of glucose and lipid metabolism, and IR‐related parameters. Conversely, they were negatively correlated with M‐values and circulating Adipoq levels. After controlling for anthropometric variables, binary logistic regression analysis revealed that serum DKK1 was significantly correlated with PCOS and IR. Our intervention study showed that circulating DKK1 was regulated by blood glucose and insulin levels, suggesting that it could be a useful biomarker for PCOS‐related metabolic morbidities.

It should be noted, however, that the diagnostic performance of DKK1 alone shows certain limitations—particularly for IR, where the sensitivity was relatively high but the specificity was modest. Several factors may contribute to this finding. First, PCOS and IR are heterogeneous conditions influenced by diverse genetic, hormonal, and metabolic backgrounds, which could reduce the discriminative power of a single biomarker. Second, the upregulation of DKK1 may not be exclusive to PCOS and IR but also occur in other metabolic disorders characterized by chronic inflammation and IR, thereby diminishing its diagnostic specificity. Taken together, these findings suggest that circulating DKK1 may not serve as a standalone diagnostic marker; nevertheless, its high sensitivity confers a strong negative predictive value, indicating that DKK1 could be a useful adjunct biomarker for screening or ruling out PCOS‐related metabolic morbidities in clinical settings.

Since more than 70% of PCOS patients have IR and compensatory hyperinsulinemia, identifying biomarkers for PCOS‐related metabolic disorders and IR is crucial for diagnosis and prognosis [21]. A recent study showed that HMGB1 and DKK1 are higher in T2DM, leading to deficits in β‐cell function and increased glucose toxicity [22]. As a specific antagonist of the Wnt signaling pathway, DKK1 is potential in mediating dysfunctional glucolipid metabolism and IR, which is also a significant drug target in treating T2DM [23]. A previous study identified DKK1 as a PCOS‐related gene regulated by chromobox homolog 2 (CBX2) [24]. In addition, another study found that mutations in the DKK1 gene lead to high androgen metabolism characteristics in patients with PCOS [25]. Consistent with these, our study found that circulating DKK1 levels may be a better indicator of glucose and lipid metabolism disorders in PCOS women. We also used the EHC test to evaluate the relationship between DKK1 and IR and found that DKK1 levels could reflect the IR status in PCOS individuals. We observed that in women with PCOS and IR, circulating DKK1 levels were regulated by blood glucose and insulin levels, while in normal women, OGTT and EHC tests did not result in alterations to DKK1 levels. Interestingly, in the state of high glucose and insulin, serum DKK1 in PCOS patients slightly increased, while in IR individuals, it significantly decreased. We speculate that this discrepancy may be due to several factors, including small sample deviation, the influence of sex hormones, or depletion of DKK1‐secreting cells caused by long‐term hyperinsulinemia.

Our study found that in PCOS and IR individuals, circulating DKK1 and Adipoq levels showed an opposite change, with a negative correlation between the two. Yang et al. found that DKK1 can promote fatty acid uptake in hepatocytes, exacerbate fat metabolism disorders, and promote IR and non‐alcoholic fatty liver disease (NAFLD) [17]. Furthermore, current findings have suggested that higher levels of serum DKK1 may be associated with greater skeletal muscle adiposity, which is a fact that emerging evidence indicates that ectopic skeletal muscle adiposity may be a risk factor for type 2 diabetes [7]. We therefore speculate that DKK1 may act as a negative regulator of metabolic disorders and insulin sensitivity.

Adipoq is an adipocytokine related to insulin sensitivity, and its levels decrease in the presence of IR [26]. Studies have shown that circulating Adipoq levels decrease at IR and increase again after insulin sensitivity recovery [27]. Our study found that in PCOS and IR individuals, circulating DKK1 and Adipoq levels showed an opposite change, with a negative correlation between the two. We therefore speculate that DKK1 may act as a negative regulator of metabolic disorders and insulin sensitivity. High levels of DKK1 may indicate severe IR and metabolic disorders in PCOS women. However, this hypothesis requires further validation through large‐scale clinical and basic research.

While our study has made a new discovery regarding the significance of circulating DKK1 levels in PCOS and metabolic disorders, it is subject to some limitations. First, our research population was Han people, and the results may not apply to other races. Moreover, our study is cross‐sectional, and long‐term changes in circulating DKK1 levels cannot be observed. We also cannot determine the causal relationship between circulating DKK1 and the occurrence and development of PCOS and IR. Finally, our study findings are limited to PCOS patients living with obesity, necessitating further research to explore the variations in circulating DKK1 levels among lean PCOS patients. In conclusion, our study underscores the substantial elevation in DKK1 levels in PCOS patients and the potential relationship between DKK1 and PCOS and metabolic disorders. Our results propose that DKK1 may function as a useful biomarker for PCOS‐related metabolic morbidities, although the physiological and pathological significance of DKK1 changes.

## 5 Conclusions

In conclusion, our study highlights the significant increase in DKK1 levels in PCOS patients and reveals the potential relationship between DKK1 and PCOS and metabolic disorders. We also evaluated the regulatory factors of circulating DKK1 by blood glucose and insulin levels. We propose that DKK1 could be a biomarker associated with PCOS and metabolic disorders. Nevertheless, the physiological and pathological implications of DKK1 fluctuations warrant further investigation.

## Author Contributions

**Jiaxiu Ling:** writing – original draft. **Hao Wang:** software. **Rui Liu:** investigation. **Juan Xiao:** formal analysis. **Sheng Qiu:** formal analysis. **Xiaotian Lei:** formal analysis. **Mengliu Yang:** data curation. **Yerui Lai:** formal analysis. **Hua Huang:** writing – review and editing. **Zerong Liang:** writing – review and editing.

## Funding

This work was supported by the Joint Project of Chongqing Health Commission and Science and Technology Bureau, 2021MSXM283, 2024MSXM148; Postgraduate research and innovation projects of Chongqing Municipal Education Commission, CYS21221; Natural Science Foundation Project of Chongqing, CSTB2022NSCQ‐MSX1522; Science and Technology Program of Health Bureau of Chongqing, 2016MSXM083 and 2017MSXM20; China Postdoctoral Science Foundation, 2022MD713711; National Natural Science Foundation of China, 82000792, 82100852; Chongqing Nature Science Foundation Project‐Postdoctoral Science Foundation Project, 2022NSCQ‐BHX0541.

## Ethics Statement

This study was approved by the Human Research Ethics Committee of Chongqing Medical University (2014 Ethical Review No. 72) and registered at the Chinese Clinical Trial Registry (ChiCTR2000032878). Animal experiments were approved by the Animal Studies Committee of The Second Affiliated Hospital of Chongqing Medical University (IACUC‐SAHCQMU‐2024‐00072) and were consistent with the National Institutes of Health Guide for the Care and Use of Laboratory Animals (NIH Publications No. 8023, revised 1978).

## Conflicts of Interest

The authors declare no conflicts of interest.

## Data Availability

Data will be made available on request.
